# Development and Validation of a Prognostic Autophagy-Related Gene Pair Index Related to Tumor-Infiltrating Lymphocytes in Early-Stage Lung Adenocarcinoma

**DOI:** 10.3389/fcell.2021.719011

**Published:** 2021-09-20

**Authors:** Zi-Hao Wang, Yu Li, Pei Zhang, Xuan Xiang, Xiao-Shan Wei, Yi-Ran Niu, Lin-Lin Ye, Wen-Bei Peng, Si-Yu Zhang, Qian-Qian Xue, Qiong Zhou

**Affiliations:** Department of Respiratory and Critical Care Medicine, Union Hospital, Tongji Medical College, Huazhong University of Science and Technology, Wuhan, China

**Keywords:** autophagy, autophagy-related genes, gene signatures, lung adenocarcinoma, prognosis, tumor-infiltrating lymphocytes

## Abstract

The role of autophagy in lung cancer is context-dependent and complex. Recent studies have reported the important role of autophagy in tumor immune escape. However, the association between autophagy and tumor-infiltrating lymphocytes (TILs) in early-stage lung adenocarcinoma (LUAD) remains unclear. In this study, we aimed to develop and validate the autophagy-related gene pair index (ATGPI) and autophagy clinical prognostic index (ACPI) in multiple LUAD cohorts, including The Cancer Genome Atlas (TCGA) cohort, Gene Expression Omnibus cohorts, and one cohort from Union Hospital, Wuhan (UH cohort), using a Cox proportional hazards regression model with the least absolute shrinkage and selection operator. Multivariate Cox regression analysis demonstrated that there was a significant difference in overall survival (OS) between patients with high and low ATGPI in the testing [hazard ratio (HR) = 1.97; *P* < 0.001] and TCGA validation (HR = 2.25; *P* < 0.001) cohorts. Time-dependent receiver operating characteristic curve analysis was also performed. We found that high ATGPI could accurately identify patients with early-stage LUAD with shorter OS, with the areas under the curve of 0.703 and 0.676 in the testing and TCGA validation cohorts, respectively. Concordance index (C-index) was used to evaluate the efficiency of ATGPI and ACPI. The C-index of ACPI was higher than that of ATGPI in the testing (0.71 vs. 0.66; *P* < 0.001), TCGA validation (0.69 vs. 0.65; *P* = 0.028), and UH (0.80 vs. 0.70; *P* = 0.015) cohorts. TIL analysis demonstrated that the proportions of tumor-infiltrating CD4^+^ T cells were lower in the high-ATGPI group than in the low-ATGPI group in both the TCGA validation and UH cohorts. These results indicate the potential clinical use of ATG signatures which are associated with TILs, in identifying patients with early-stage LUAD with different OS.

## Introduction

Macroautophagy (referred to here as autophagy) is a highly conserved catabolic process that involves the formation of autophagosomes, growth of double membranes, merging with lysosomes, and disintegration of engulfed cellular proteins and organelles (Levine and Kroemer, 2008; Levy et al., 2017). Autophagy plays a context-dependent role in cancer development. The enhancement and inhibition of autophagy have both been suggested as therapeutic strategies in cancers (Levy and Thorburn, 2011; Amaravadi et al., 2016). The dynamic process of autophagy in cancers is coordinated by a series of proteins encoded by autophagy-related (ATG) genes and their various activation factors such as the tumor type and status of oncogenes and tumor suppressors (Kimmelman and White, 2017). The role of autophagy in lung cancer has been previously demonstrated in multiple genetically engineered mouse models. Inactivation of the essential autophagy gene *ATG5* accelerates the early phases of non-small cell lung cancer (NSCLC) oncogenesis (Rao et al., 2014). Additionally, autophagy maintains the functioning of mitochondria to support the metabolism and growth of *KRAS*-driven NSCLC (Guo and White, 2013). Moreover, multiple factors, including circular RNA, REV-ERB agonists, and copper, can function as autophagy regulators to modulate the proliferation, migration, and invasion of lung cancer (Chen et al., 2020; Shen et al., 2020; Tsang et al., 2020). These findings demonstrate the biological significance of autophagy in lung cancer. However, few studies have focused on the potential of ATG genes in predicting survival of patients with early-stage lung adenocarcinoma (LUAD).

Lung cancer is the most frequently diagnosed cancer and the most common cause of cancer-related mortality worldwide, and LUAD is the most common histologic subtype of NSCLC (Torre et al., 2015). LUAD is currently considered a cluster of discrete molecular subtypes that are defined by high-throughput genomic profiling. Therefore, it is crucial to assess the complex genomic alterations of LUAD using next-generation sequencing technologies The Cancer Genome Atlas Research Network (2014). Combining sequencing profiles with clinical characteristics will improve the prognostic evaluation of NSCLC. To date, multiple prognostic biomarkers, including long non-coding RNA signatures, microRNA signatures, circulating tumor DNA signatures, DNA methylation signatures, and immune signatures, have been constructed for NSCLC (Ajona et al., 2013; Hao et al., 2017; Li B. et al., 2017; Li Y. et al., 2017; Ooki et al., 2017; Martínez-Terroba et al., 2018; Dong et al., 2019). However, the clinical application of these signatures is limited because of technical biases across different platforms.

Thus, in this study, we aimed to develop and validate the autophagy-related gene pair index (ATGPI) and autophagy clinical prognostic index (ACPI) in multiple LUAD cohorts. ATGPI and ACPI could accurately distinguish early-stage LUAD with different overall survival (OS) in independent cohorts. Compared with ATGPI, the combination with clinical characteristics improved the accuracy of ACPI. The proportions of tumor-infiltrating CD4^+^ T cells were lower in the high-ATGPI group than in the low-ATGPI group.

## Materials and Methods

### Data Collection and LUAD Cohorts

Gene expression data from 13 public LUAD datasets and one LUAD cohort from Union Hospital, Wuhan, China were used in this retrospective study (Bhattacharjee et al., 2001; Beer et al., 2002; Takeuchi et al., 2006; Kuner et al., 2009; Tomida et al., 2009; Okayama et al., 2012; Wilkerson et al., 2012; Rousseaux et al., 2013; Sato et al., 2013; Tang et al., 2013; Der et al., 2014). In total, 68 samples of LUAD tissues were obtained from surgical resections of patients without preoperative treatment from Union Hospital, Wuhan, China. Specimens were collected from July 2017 to August 2020 and were stored at −80°C. Human specimens were obtained with the consent of patients; the study was approved by Ethics Committees of Tongji Medical College, Huazhong University of Science and Technology (2019-S671). RNA-Seq data and clinical information of LUAD patients were downloaded from Genomic Data Commons Data Portal^1^. Data from 12 datasets (GSE11969, GSE13213, GSE14814, GSE26939, GSE30219, GSE31210, GSE31547, GSE37745, GSE41271, GSE42127, GSE50081, and GSE68465) were downloaded from the Gene Expression Omnibus (GEO)^2^. Patients who received neoadjuvant therapy, adjuvant chemotherapy, or other immune-modulating therapy were excluded. All microarray datasets were merged into one dataset and then randomly divided into training and testing cohorts. The Cancer Genome Atlas (TCGA) and Union Hospital (UH) cohorts were included as independent validation cohorts. The information about all platforms included in this study is shown in Table 1. Raw median intensity values of tumor samples in Agilent datasets were extracted using the R package agilp (v3.4.0) (Stafford and Brun, 2007). For Affymetrix microarrays, gene expression profiles were normalized using the R package affy (version 1.50.0) (Gautier et al., 2004). For Illumina datasets, model-based background correction processed data were downloaded.

**TABLE 1:** Datasets used in this study.

**Datasets**		**Platform**	**No. of enrolled patients**
Training testing	GSE11969	Agilent Homo sapiens 21.6K custom array	65
	GSE13213	Agilent-014850 whole human genome microarray 4 × 44K G4112F	92
	GSE14814	Affymetrix human genome U133A array	70
	GSE26939	Agilent-UNC-custom-4 × 44K	60
	GSE30219	Affymetrix human genome U133 Plus 2.0 array	82
	GSE31210	Affymetrix human genome U133 plus 2.0 array	226
	GSE31547	Affymetrix human genome U133A array	24
	GSE37745	Affymetrix human genome U133 plus 2.0 array	89
	GSE41271	Illumina Humanwg-6 v3.0 expression beadchip	125
	GSE42127	Illumina Humanwg-6 v3.0 expression beadchip	109
	GSE50081	Affymetrix human genome U133 plus 2.0 array	127
	GSE68465	Affymetrix human genome U133A array	369
Validation	TCGA	Illumina HiSeq	382
Validation	UH cohort	–	68

### Construction of ATGPI as a Robust Prognostic Biomarker for Early-Stage LUAD

The method used to construct the gene pair index was based on that used in a previous study (Li B. et al., 2017). The ATG genes were downloaded from The Human Autophagy Database^3^. Among them, only 142 ATG genes detected in all the datasets were included. To construct the autophagy-related gene pairs (ATGPs), the ATG genes were compared in pairs. To construct one gene pair, the ATGP score was 0 if the gene expression level of the ATG gene1 was more than that of the ATG gene2; otherwise, the ATGP score was 1. The ATGPs with constant values (0 or 1) in each dataset were excluded considering the probable biases. The log-rank test was used to select ATGPs that were associated with the OS of patients. ATGPs with *p* values < 0.05 were included in ATGPI in the training cohort. A Cox proportional hazards regression model with the least absolute shrinkage and selection operator (LASSO) was used to select appropriate ATGPs. The optimal model parameter λ was evaluated by 10-fold cross-validation at 1 standard error as previously recommended. The final ATGPI was constructed using 10 ATGPs that contained 15 unique ATG genes. Complete information of the final 10 ATGPs is provided in Table 2. Time-dependent receiver operating characteristic (ROC) curves were used to determine the optimal cutoff of ATGPI. The nearest neighbor estimation method was used to estimate the ROC curve. Patients were divided into low- and high-risk groups based on the optimal cutoff value of ATGPI.

**TABLE 2:** The autophagy-related (ATG) genes used to construct the autophagy-related gene pair index (ATGPI).

**ATG gene^a^ 1**	**Full name**	**ATG gene 2**	**Full name**	**ATGP^b^**	**Coefficient**
*ARSB*	Arylsulfatase B	*BIRC5*	Baculoviral IAP repeat-containing 5	*ARSB – BIRC5*	0.2155943
*BAK1*	BCL2-antagonist/killer 1	*CX3CL1*	Chemokine (C-X3-C motif) ligand 1	*BAK1 – CX3CL1*	−0.051255
*BAX*	BCL2-associated X protein	*CX3CL1*	Chemokine (C-X3-C motif) ligand 1	*BAX – CX3CL1*	−0.199284
*BCL2*	B-cell CLL/lymphoma 2	*SPHK1*	Sphingosine kinase 1	*BCL2 – SPHK1*	0.0516312
*BIRC5*	Baculoviral IAP repeat-containing 5	*MAP2K7*	Mitogen-activated protein kinase kinase 7	*BIRC5 – MAP2K7*	−0.225825
*BIRC5*	Baculoviral IAP repeat-containing 5	*TSC1*	Tuberous sclerosis 1	*BIRC5 – TSC1*	−0.142442
*CCL2*	Chemokine (C-C motif) ligand 2	*CX3CL1*	Chemokine (C-X3-C motif) ligand 1	*CCL2 – CX3CL1*	−0.275192
*CCR2*	Chemokine (C-C motif) receptor 2	*SPHK1*	Sphingosine kinase 1	*CCR2 – SPHK1*	0.3833014
*CDKN2A*	Cyclin-dependent kinase inhibitor 2A	*MAPK9*	Mitogen-activated protein kinase 9	*CDKN2A – MAPK9*	−0.319519
*FADD*	Fas (TNFRSF6)-associated via death domain	*HSPB8*	Heat shock 22kDa protein 8	*FADD – HSPB8*	−0.151957

*^*a*^ATG gene, autophagy-related gene.*

*^*b*^ATGP, autophagy-related gene pair.*

### Validation of the Prognostic Value of ATGPI

To assess the prognostic value of ATGPI, univariate Cox regression analysis was performed in the training, testing, and TCGA validation cohorts. Age and tumor stage were considered continuous variables. The tumor stage was considered as I = 1 and II = 2. Male and high ATGPI were risk factors in the univariate analysis. The prognostic accuracy of ATGPI was assessed using time-dependent ROC curves.

### Association Between ATGPI and Immune Infiltration

To explore the association between ATGPI and immune infiltration, we used the ImmuCellAI method^4^ (Miao et al., 2020) to analyze tumor-infiltrating lymphocytes (TILs) abundance in the TCGA validation cohort. The proportions of TILs were compared between the high- and low-ATGPI groups.

### Combination of ATGPI and Clinical Characteristics

Multivariate Cox regression analysis was conducted by combining ATGPI with clinical factors that were significantly associated with OS in the univariate analysis. ATGPI, age, and tumor stage were combined to fit a Cox proportional hazards regression in the training cohort. The cutoff value of ACPI was estimated using time-dependent ROC curves. The prognostic accuracy of ACPI was compared with that of ATGPI using restricted mean survival (RMS) curves.

### Total RNA Isolation and Quantitative Real-Time Polymerase Chain Reaction (qRT-PCR)

Total RNAs were isolated from fresh LUAD tissues using Trizol reagent (Takara Bio Inc., China). HiScript II One Step qRT-PCR SYBR Green Kit (Vazyme, China) was used in qRT-PCR analysis. The sequences of all primers used are shown in Table 3. All primers were designed and purchased from Tsingke (Wuhan, China).

**TABLE 3:** The primer sequences of 15 autophagy-related (ATG) genes used in quantitative real-time polymerase chain reaction (qRT-PCR) analysis.

**ATG genes^a^**	**Forward primer**	**Reverse primer**
*ARSB*	CGGGAGCTCATCCACATCTC	CAATTCTGGGGGATGGGCTT
*BAK1*	ACATCCAGATGCCGGGAATG	TTCTTACTCCAGCATGGGTCC
*BAX*	CCGCCGTGGACACAGACT	TTGAAGTTGCCGTCAGAAAACA
*BCL2*	TCGCCCTGTGGATGACTGA	CAGAGACAGCCAGGAGAAATCA
*BIRC5*	AGAACTGGCCCTTCTTGGAGG	CTTTTTATGTTCCTCTATGGGGTC
*CCL2*	GAAAGTCTCTGCCGCCCTT	ACAGATCTCCTTGGCCACAA
*CCR2*	GTTCTCTTCCTGACCACCTTC	CTTCGGAACTTCTCACCAACA
*CDKN2A*	TGGTTGCCAGGACGAATTGA	AAGATACGAGATCCCGCTGC
*FADD*	CACCACGGTGTGACGAAATG	ATCTGCTTCTCGAAGGTGCC
*CX3CL1*	GGATGCAGCCTCACAGTCCTTAC	GGCCTCAGGGTCCAAAGACA
*SPHK1*	CTTGCAGCTCTTCCGGAGTC	GCTCAGTGAGCATCAGCGTG
*MAP2K7*	CCTCCCTGGAACAGAAGCTG	AGGAGCAGGGCTTAGAGTGA
*TSC1*	CCGTGGCCCTATGCTTGTAA	CGGCTTTGCCCACATATTCG
*MAPK9*	AGTCATCCTGGGTATGGGCT	GGAAGGATACGGTCAGTGCC
*HSPB8*	GCTTCAAGCCAGAGGAGTTG	ACTGTCACAGGATCCACCTCT

*^*a*^ATG genes, autophagy-related genes.*

### Immunohistochemical (IHC) Analyses

The tumor tissues collected from LUAD patients were fixed, dehydrated, and embedded in paraffin. The 4-μm-thick sections were then stained for IHC analyses using recombinant anti-CD4 antibodies (ab133616, Abcam, United Kingdom).

### Statistical Analysis

The log-rank test was used to select ATGPs associated with OS. The LASSO Cox proportional hazards regression model was used to select ideal prognostic ATGPs to construct ATGPI. The time-dependent ROC curve was used to determine the optimal cutoff of ATGPI and ACPI. The discrimination of the model was evaluated using the areas under the curve (AUCs). The Kaplan–Meier method was used to estimate survival curves between the low- and high-risk groups. A multivariate Cox proportional hazards regression model that included variates significantly associated with OS in the univariate analysis was used. The concordance index (C-index) was calculated using survcomp (version 1.22.0) and was compared using compareC (version 1.3.1) packages (Kang et al., 2015). The RMS curve and RMS time ratio were estimated using survival (version 2.44-1.1) and survRM2 (version 1.0-2) packages. All statistical analyses were performed using R (version 3.6.1), *P* values < 0.05 were considered statistically significant.

## Results

### Clinical Characteristics of Patients

**FIGURE 1:**
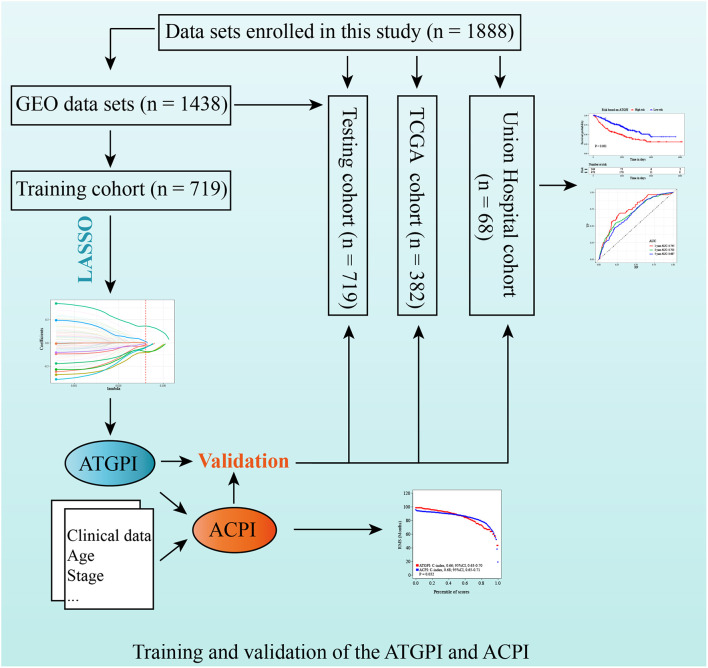
Study design for the construction and validation of the autophagy-related gene pair index (ATGPI) and autophagy clinical prognostic index (ACPI) in early-stage lung adenocarcinoma.

Detailed patient characteristics are shown in Table 4. A total of 1888 LUAD patients from multiple datasets were included. No statistically significant difference was observed between training and testing cohorts with respect to clinical characteristics. The analysis process of this study was demonstrated in a flow chart (Figure 1).

**TABLE 4:** Clinical characteristics of patients enrolled in this study.

**Characteristics**	**Training cohort**	**Testing cohort**	*P* value*	**TCGA cohort**	**UH cohort**
			
	***n* = 719**	***n* = 719**		***n* = 382**	***n* = 68**
Median age (Interquartile range)	62 (56–70)	64 (57–70)	0.033	67 (59–72)	61 (54–68)
Gender					
Female (%)	355 (49.4)	361 (50.2)	0.752	205 (53.7)	42 (61.8)
Male (%)	364 (50.6)	358 (49.8)	-	177 (46.3)	26 (38.2)
Stage					
I (%)	557 (77.5)	545 (75.8)	0.455	262 (68.6)	39 (57.4)
II (%)	162 (22.5)	174 (24.2)	–	120 (31.4)	29 (14.7)
Median overall survival in days	1704	1650	0.23	701.5	257
No. of death (%)	268 (37.3)	284 (39.5)	0.386	118 (30.9)	13 (19.1)

*The difference between training and testing cohorts was calculated.

*Age was compared with Wilcoxon rank-sum test; gender and stage were compared with chi-squared test; follow-up difference was assessed with log-rank test.*

### Construction of ATGPI

**FIGURE 2:**
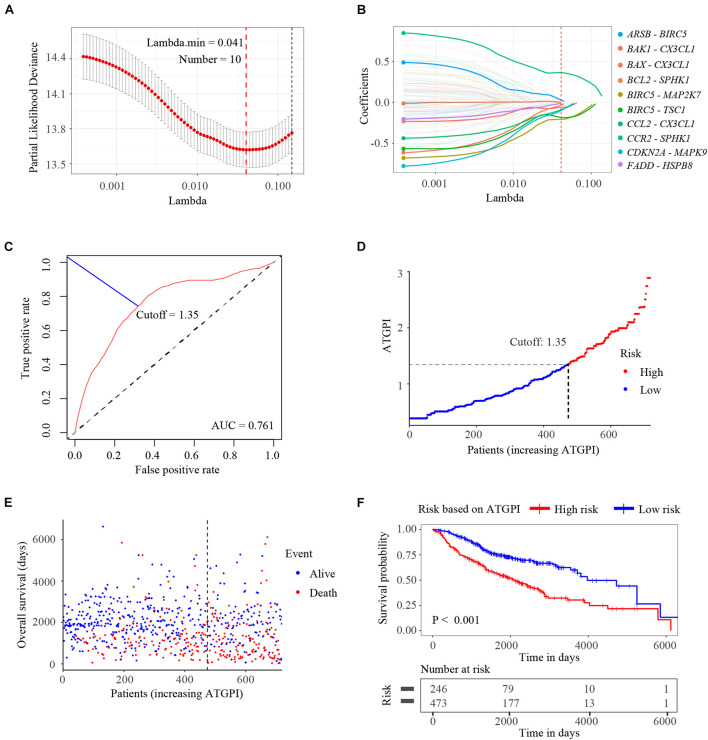
Construction of the autophagy-related gene pair index (ATGPI), a classifier comprising 15 unique autophagy-related genes. **(A)** The penalty parameter was estimated by 10-fold cross-validation in the training data set at 1 SE beyond the minimum partial likelihood deviance. **(B)** The LASSO coefficient profiles of the 10 prominent autophagy-related gene pairs. **(C)** The optimal cutoff of the ATGPI for stratifying patients based on time-dependent receiver operator characteristic curve analysis in the training cohort. **(D)** The risk curve of the ATGPI in the training cohort. **(E)** The scatterplot of vital status of each patient in the training cohort. **(F)** The Kaplan-Meier survival curves of low and high ATGPI groups.

Among the ATG genes from The Human Autophagy Database, 142 ATG genes were detected in all public datasets enrolled in this study. A total of 10011 ATGPs were built. Among 5402 ATGPs with differential expression in all datasets, 96 ATGPs that were associated with OS in the training dataset were selected to fit the LASSO regression model. Finally, 10 ATGPs that contained 15 unique ATG genes were selected to construct the ATGPI (Figures 2A,B). We then built the ATGPI using Cox proportional hazards regression. Based on the time-dependent ROC curve analysis, the optimal cutoff value for ATGPI was 1.35 (Figure 2C). Risk curve and scatterplot were used to determine ATGPI and the vital status of each patient. Patients in the high-ATGPI group had higher mortality than those in the low-ATGPI group (Figures 2D,E). Survival curves of different groups were estimated using the Kaplan–Meier method and were compared using the log-rank test (*P* < 0.001) (Figure 2F).

### Validation of ATGPI in the Testing and TCGA Cohorts

**FIGURE 3:**
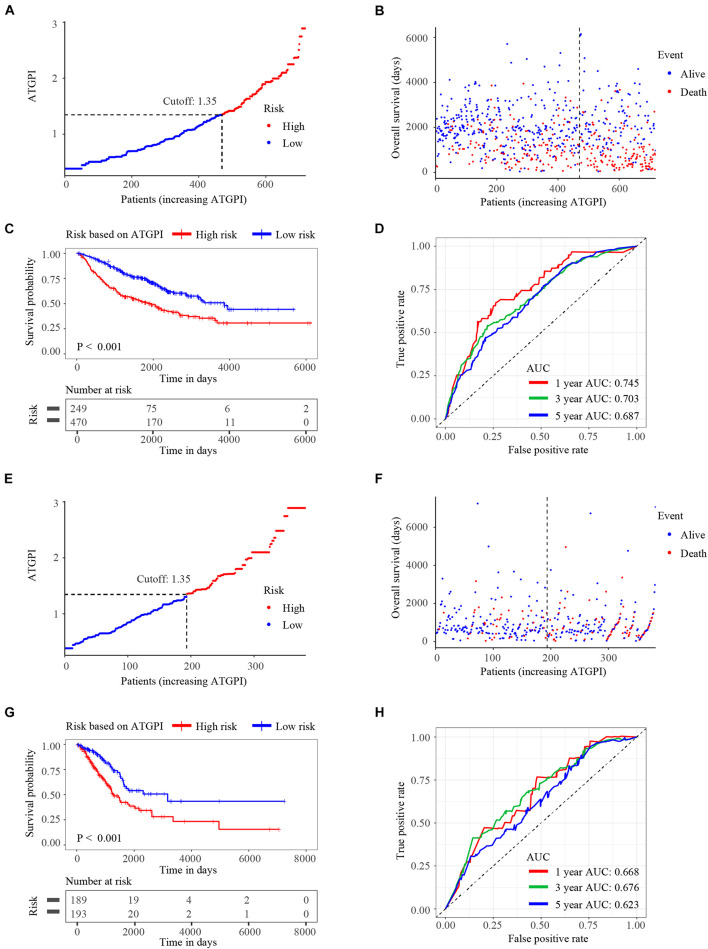
The risk curves, Kaplan-Meier (KM) curves, and time-dependent receiver operator characteristic (ROC) curves of patients in the testing and The Cancer Genome Atlas (TCGA) validation cohorts based on the autophagy-related gene pair index (ATGPI). **(A,B)** Risk curves and scatterplots of vital status for patients in the testing cohort. **(C)** The KM curves of low and high ATGPI groups in the testing cohort. **(D)** The area under the curves (AUC) of time-dependent ROC curves in the testing cohort. **(E,F)** Risk curves and scatterplots of vital status for patients in the TCGA validation cohort. **(G)** The KM curves of low and high ATGPI groups in the TCGA validation cohort. **(H)** The AUC of time-dependent ROC curves in the TCGA validation cohort.

Autophagy-related gene pair index in the testing and TCGA validation cohorts was calculated using the same model in the training cohort. ATGPI and the vital status of each patient with LUAD were demonstrated by risk curves and scatterplots in the testing (Figures 3A,B) and validation (Figures 3E,F) cohorts. The Kaplan–Meier method and log-rank test demonstrated that ATGPI stratified early-stage LUAD into different prognostic groups in the testing (*P* < 0.001) (Figure 3C) and TCGA validation (*P* < 0.001) (Figure 3G) cohorts. In the testing cohort, the AUCs at 1, 3, and 5 years were 0.745, 0.703, and 0.687, respectively (Figure 3D). In the TCGA validation cohort, the AUCs at 1, 3, and 5 years were 0.668, 0.676, and 0.623, respectively (Figure 3H).

### ATGPI as an Independent Prognostic Biomarker Related to Immune Infiltration for Early-Stage LUAD

**FIGURE 4:**
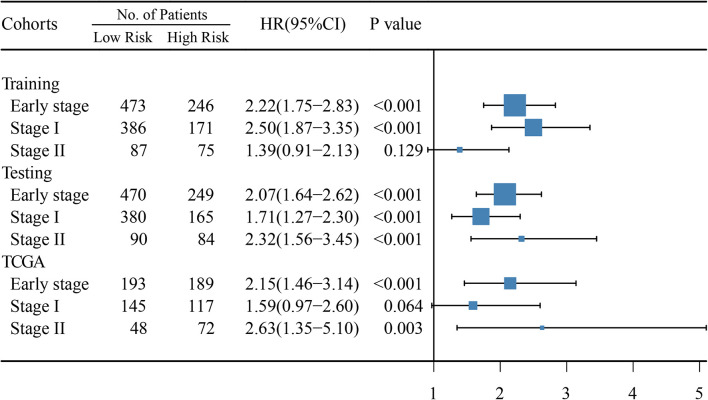
Hazard ratio (HR) between high- and low-risk groups based on the autophagy-related gene pair index (ATGPI) was evaluated using univariate cox proportional hazards regression. The length of the horizontal line corresponds to the confidence interval, and the size of the blue square is proportional to the number of patients. The vertical line indicates that HR is equal to 1.

**FIGURE 5:**
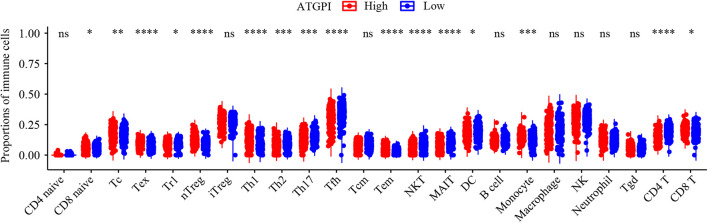
The immune infiltration status of high- and low-autophagy-related gene pair index (ATGPI) groups in the TCGA validation cohort. The percentages of different tumor-infiltrating lymphocytes (TILs) were compared by Wilcoxon rank sum test. ns, *P* ≥ 0.05; *P < 0.05; **P < 0.01; ***P < 0.001; ****P < 0.0001.

In early-stage (stage I and II) LUAD, ATGPI stratified patients into different prognostic groups in the testing [hazard ratio (HR) = 2.07; 95% confidence interval (CI), 1.64–2.62; *P* < 0.001] and TCGA validation (HR = 2.15; 95% CI, 1.46–3.14; *P* < 0.001) cohorts. In stage II LUAD, ATGPI retained its high prognostic accuracy in the testing (HR = 2.32; 95% CI, 1.56–3.45; *P* < 0.001) and the TCGA validation (HR = 2.63; 95% CI, 1.35–5.10; *P* = 0.003) cohorts (Figure 4). Multivariate analysis was performed with variables that were significantly associated with OS in the univariate analysis, including age, ATGPI, and stage. Multivariate Cox proportional hazards regression analyses revealed that high ATGPI was an independent risk factor for poor prognosis of early-stage LUAD in the training (HR = 1.95; 95%CI, 1.52–2.50; *P* < 0.001), testing (HR = 1.97; 95%CI, 1.56–2.50; *P* < 0.001) and TCGA validation (HR = 2.25; 95%CI, 1.51–3.36; *P* < 0.001) cohorts (Table 5). ImmuCellAI analysis demonstrated that the percentages of CD4^+^ T cells infiltration were significantly different between the high- and low- ATGPI groups (Figure 5). The mean level of CD4^+^ TILs in the low-ATGPI group was 1.3-fold that in the high-ATGPI group (*P* < 0.0001).

**TABLE 5:** Univariate and multivariate analyses of autophagy-related gene pair index (ATGPI) and clinical characteristics in the training, testing, and The Cancer Genome Atlas (TCGA) validation data sets.

**Cohorts**		**Variables**	**Univariate**			**Multivariate**		
			**HR**	**95%CI**	***P* value**	**HR**	**95% CI**	***P* value**
Early stage (stage I and stage II)	Training	Risk	2.22	1.75–2.83	<0.001	1.95	1.52–2.50	<0.001
		Gender	1.41	1.11–1.80	0.005	1.26	0.99–1.61	0.064
		Age	1.02	1.01–1.04	<0.001	1.02	1.01–1.03	0.001
		Stage	1.89	1.46–2.44	<0.001	1.59	1.22–2.07	0.001
	Testing	Risk	2.07	1.64–2.62	<0.001	1.97	1.56–2.50	<0.001
		Gender	1.56	1.23–1.98	<0.001	1.41	1.11–1.80	0.004
		Age	1.04	1.03–1.06	<0.001	1.04	1.03–1.06	<0.001
		Stage	2.77	2.17–3.53	<0.001	2.56	2.00–3.27	<0.001
	TCGA validation	Risk	2.15	1.46–3.14	<0.001	2.25	1.51–3.36	<0.001
		Gender	1.07	0.74–1.54	0.718	–	–	–
		Age	1.02	1.00–1.04	<0.001	1.03	1.01–1.05	0.002
		Stage	2.32	1.61–3.34	<0.001	2.09	1.44–3.02	<0.001

*Age, stage, grade was coded as continuous variable. Specifically, stage was coded as I1, II = 2. The risk factors of gender, and autophagy risk are male and high risk based on ATGPI.*

### Integrated Prognostic Model by Combining ATGPI With Clinical Characteristics

**FIGURE 6:**
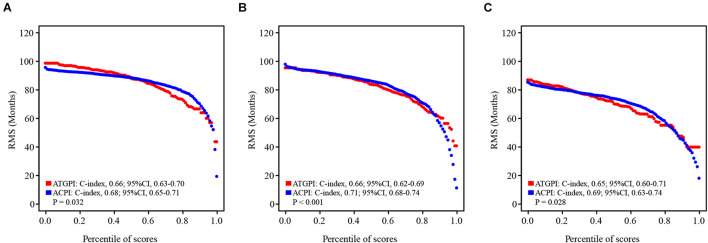
The restricted mean survival (RMS) curves for autophagy-related gene pair index (ATGPI) and autophagy clinical prognostic index (ACPI) in the training, testing, and The Cancer Genome Atlas (TCGA) validation cohorts. **(A–C)** The RMS curves for the ATGPI and ACPI in the training cohort, the testing cohort, and the TCGA validation cohort. Each point demonstrates the RMS time of corresponding ATGPI and ACPI value. The slope of RMS curves was larger, the estimation for overall survival was better. Concordance index (C-index) was used to represent the difference between the ATGPI and ACPI.

ATGPI, age, and tumor stage were independent prognostic factors for OS of patients with early-stage LUAD; thus, these variables were combined to fit a Cox proportional hazards regression model in the training cohort, and ACPI was built (ACPI = 0.6657 × ATGPI + 0.4445 × stage + 0.0199 × age). The optimal cutoff value of ACPI was determined to be 1.31 based on the time-dependent ROC curve analysis in the training cohort. The RMS curves of continuous ATGPI and ACPI demonstrated that ACPI was significantly better in predicting OS than ATGPI in the training (C-index: 0.66, 95% CI, 0.63–0.70 vs. C-index: 0.68, 95% CI, 0.65–0.71; *P* = 0.032), testing (C-index: 0.66, 95% CI, 0.62–0.69 vs. C-index: 0.71, 95% CI, 0.68–0.74; *P* < 0.001), and TCGA validation (C-index: 0.65, 95% CI, 0.60–0.71 vs. C-index: 0.69, 95% CI, 0.63–0.74; *P* = 0.028) cohorts (Figure 6).

### Validation of ATGPI and ACPI in the UH Cohort

**FIGURE 7:**
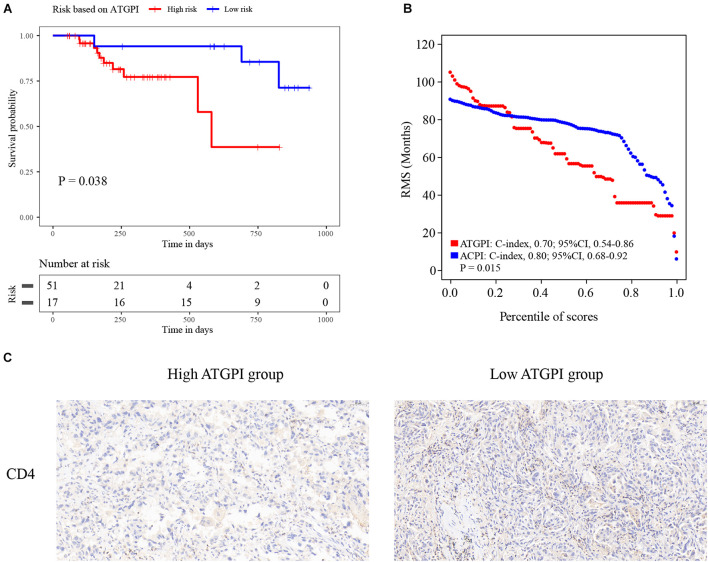
The Kaplan–Meier curves for the autophagy-related gene pair index (ATGPI) and restricted mean survival (RMS) curves for the ATGPI and autophagy clinical prognostic index (ACPI) in the Union hospital (UH) cohort. **(A)** The Kaplan–Meier curves showed that patients were stratified into different prognostic groups by the ATGPI. **(B)** The RMS curves revealed that a superior estimation for overall survival was achieved by the ACPI in the UH cohort. **(C)** Immunohistochemistry (IHC) analysis showed the proportions of tumor-infiltrating CD4^+^ T cells were higher in the low-ATGPI group.

Detailed qRT-PCR data of patients are shown in Supplementary Table 1. ATGPI and ACPI of the UH cohort were calculated using the same model as that in the training cohort. The Kaplan–Meier method and log-rank test demonstrated that high ATGPI was significantly associated with short OS of patients with early-stage LUAD in the UH cohort (*P* = 0.038) (Figure 7A). The RMS curves of continuous ATGPI and ACPI in the UH cohort demonstrated that significant improvement in predicting OS was achieved by ACPI (C-index: 0.70, 95% CI, 0.54–0.86 vs. C-index: 0.80, 95% CI, 0.68–0.92; *P* = 0.015) (Figure 7B). IHC analysis demonstrated that the proportions of tumor-infiltrating CD4^+^ T cells were higher in the low-ATGPI group that in the high-ATGPI group (Figure 7C).

## Discussion

Complete surgical resection of early-stage LUAD significantly improves prognosis, but some patients are still at a high risk of recurrence and death. Previous studies have not demonstrated a survival benefit of adjuvant therapy in patients with early-stage LUAD. To identify high-risk early-stage LUAD, clinicians urgently need reliable prognostic biomarkers. In this study, we first constructed a TIL-related prognostic biomarker based on ATGPs for patients with early-stage LUAD by combining multiple gene expression datasets. To construct a robust signature, data from 12 GEO LUAD datasets were randomly divided into training and testing datasets. TCGA and UH cohorts were included as independent validation datasets. The optimal prediction model was established using the Cox proportional hazards regression model with the LASSO in the training cohort and was validated in independent cohorts. Moreover, we integrated ATG signatures with clinical characteristics to build the ACPI, which improved the estimation of prognosis for patients with early-stage LUAD. The expression levels of genes in each sample were compared in pairs to generate the ATGPI. This gene pair-based approach was based on the relative gene expression of each sample and did not require normalization.

Autophagy is a dynamic process that captures and degrades intracellular components such as proteins and organelles to sustain metabolism and homeostasis (Mizushima and Komatsu, 2011). This survival-promoting pathway mediated by evolutionarily conserved ATG genes, recycles intracellular materials in lysosomes, prevents the accumulation of toxic cellular wastes, and involves multiple signaling pathways, such as the RAS, p53/DRAM, PI3K/AKT/mTOR, and JAK-STAT signaling pathways (Levy et al., 2017). Autophagy plays an important role in lung cancer. For example, PAQR3 enhances autophagy induced by the EGFR inhibitor erlotinib to inhibit the growth of NSCLC cells, and a combination therapy, including lipophilic bisphosphonates, facilitates autophagy, and prevents *KRAS*-driven LUAD cell proliferation (Xia et al., 2014). Because of the important role of autophagy in lung cancer, including ATG gene signatures in survival prediction for lung cancer is crucial.

To construct a robust ATG signature, we identified ATG genes from The Human Autophagy Database and profiled the mRNA expression of 142 ATG genes that were detected in all datasets used in this study. The final ATGPI was constructed using 10 ATGPs consisting of 15 ATG genes (*ARSB*, *BAK1*, *BAX*, *BCL2*, *BIRC5*, *CCL2*, *CCR2*, *CDKN2A*, *FADD*, *CX3CL1*, *SPHK1*, *MAP2K7*, *TSC1*, *MAPK9*, and *HSPB8*). Dysregulation of the expression of anti-apoptotic BCL-2 family proteins is observed in multiple cancers. Combining BCL-2/BCL-XL inhibitors with kinase inhibitors enhanced the efficacy of EGFR inhibitors against *EGFR* mutant LUAD (Gong et al., 2007). Lung cancer cells can induce macrophage infiltration by increasing the production of CCL2 and CXCL3 (Schmall et al., 2015). Inhibition of the CCL2-CCR2 signaling pathway blocks the recruitment of inflammatory monocytes and prolongs survival in a mouse model (Qian et al., 2011). Increased expression of *FADD* is associated with adverse clinical outcomes in patients with LUAD (Chen et al., 2005). *SPHK1* regulates tumor cell apoptosis and promotes NSCLC development (Ma et al., 2021). Loss of *TSC1* synergizes with *KRAS* mutation to enhance the development of lung cancer in mice (Liang et al., 2010). Inactivation of *MAP2K7* in *KRAS*-driven lung cancer accelerates tumorigenesis and reduces survival (Schramek et al., 2011). The autophagy process in the NSCLC microenvironment can be modulated by variable immune signals, such as extracellular damage-associated molecular patterns and cytokines. For example, the activation of Toll-like receptors (TLR3 and TLR4) with polyinosinic:polycytidylic acid and lipopolysaccharide can induce autophagy and upregulate metastasis-related cytokines (Zhan et al., 2014). Few studies focused on the influence of the relative expression of ATGPs on cancer progression. For example, overexpression of *SPHK1* induced the expression of *BCL2* to promote the proliferation and migration of NSCLC cells, suggesting that the expression of *SPHK1* is positively associated with the expression of BCL2 (Ma et al., 2021). Additionally, the coexpression of *CCL2* and *CX3CL1* was strongly associated with prognosis of patients with soft tissue sarcoma, particularly female patients (Kehlen et al., 2014). Further studies are needed to explore the potential value of the relative expression of ATGP signatures. Bioinformatic and IHC analyses indicated that the proportions of CD4^+^ TILs were lower in the high-ATGPI group than in the low-ATGPI group. The increased infiltration of CD4^+^ TILs was associated with better prognosis of patients with esophageal squamous cell carcinoma (Chen et al., 2017) and triple-negative breast cancer (Gao et al., 2020). Furthermore, CD4^+^ TILs demonstrated a functional heterogeneity in NSCLC (Oja et al., 2018). The vital role of these ATG genes in the development and progression of NSCLC and the association between ATGPI and TILs might explain the robust prediction ability of ATGPI and ACPI.

## Limitations

Although batch effects were reduced by the pairwise comparison of ATG genes, some were unavoidable because of the combination of different platforms (Leek et al., 2010). Additionally, sampling bias caused by intratumor genetic heterogeneity was inevitable for gene expression-based signatures.

In summary, ATGPI is a prospective prognostic biomarker for LUAD, especially early-stage LUAD. Combining ATGPI with clinical characteristics improved the accuracy of the model. Further studies are needed to validate the prediction accuracy of ATGPI and ACPI and explore the clinical utility of ATGPI and ACPI in the individualized management of LUAD.

## Data Availability Statement

The datasets presented in this study can be found in online repositories. The names of the repository/repositories and accession number(s) can be found in the article/Supplementary Material.

## Ethics Statement

The studies involving human participants were reviewed and approved by the Ethics Committees of Tongji Medical College, Huazhong University of Science and Technology (2019-S671). The patients/participants provided their written informed consent to participate in this study. Written informed consent was obtained from the individual(s) for the publication of any potentially identifiable images or data included in this article.

## Author Contributions

QZ conceived the idea, designed, and supervised the study, had full access to all data and took responsibility for the integrity of the data. X-SW, YL, L-LY, W-BP, S-YZ, and Y-RN recorded and sorted the data. XX and Q-QX analyzed the data. Z-HW and PZ interpreted the data and wrote the manuscript. All authors contributed significantly to this work, agreed to be accountable for the work, and read and approved the final manuscript.

## Conflict of Interest

The authors declare that the research was conducted in the absence of any commercial or financial relationships that could be construed as a potential conflict of interest.

## Publisher’s Note

All claims expressed in this article are solely those of the authors and do not necessarily represent those of their affiliated organizations, or those of the publisher, the editors and the reviewers. Any product that may be evaluated in this article, or claim that may be made by its manufacturer, is not guaranteed or endorsed by the publisher.

## Footnotes

1
https://portal.gdc.cancer.gov/

2
https://www.ncbi.nlm.nih.gov/geo/

3
http://www.autophagy.lu/

4
http://bioinfo.life.hust.edu.cn/ImmuCellAI#!/

## Abbreviations

ATG: autophagy-related
ATGPs: autophagy-related gene pairs
ATGPI: autophagy-related gene pair index
ACPI: autophagy clinical prognostic index
AUC: area under the curve
C-index: concordance index
FP: false positive rate
GEO: gene expression omnibus
LASSO: least absolute shrinkage and selection operator
LUAD: lung adenocarcinoma
NSCLC: non-small cell lung cancer
OS: overall survival
ROC: receiver operating characteristics
TCGA: The Cancer Genome Atlas
TP: true positive rate
TILs: tumor-infiltrating lymphocytes.
