# Supplementary material for: Development and Validation of a Prognostic Autophagy-Related Gene Pair Index Related to Tumor-Infiltrating Lymphocytes in Early-Stage Lung Adenocarcinoma
Source: Front Cell Dev Biol. 2021 Sep 20;9:719011. doi: 10.3389/fcell.2021.719011 (PMC8488280; doi:10.3389/fcell.2021.719011)
Supplement: Supplementary file 1 [file Image_1.pdf]

**Supplementary Figure 1.** Quantitative real-time polymerase chain reaction (qRT-PCR) data of patients in the Union Hospital cohort.

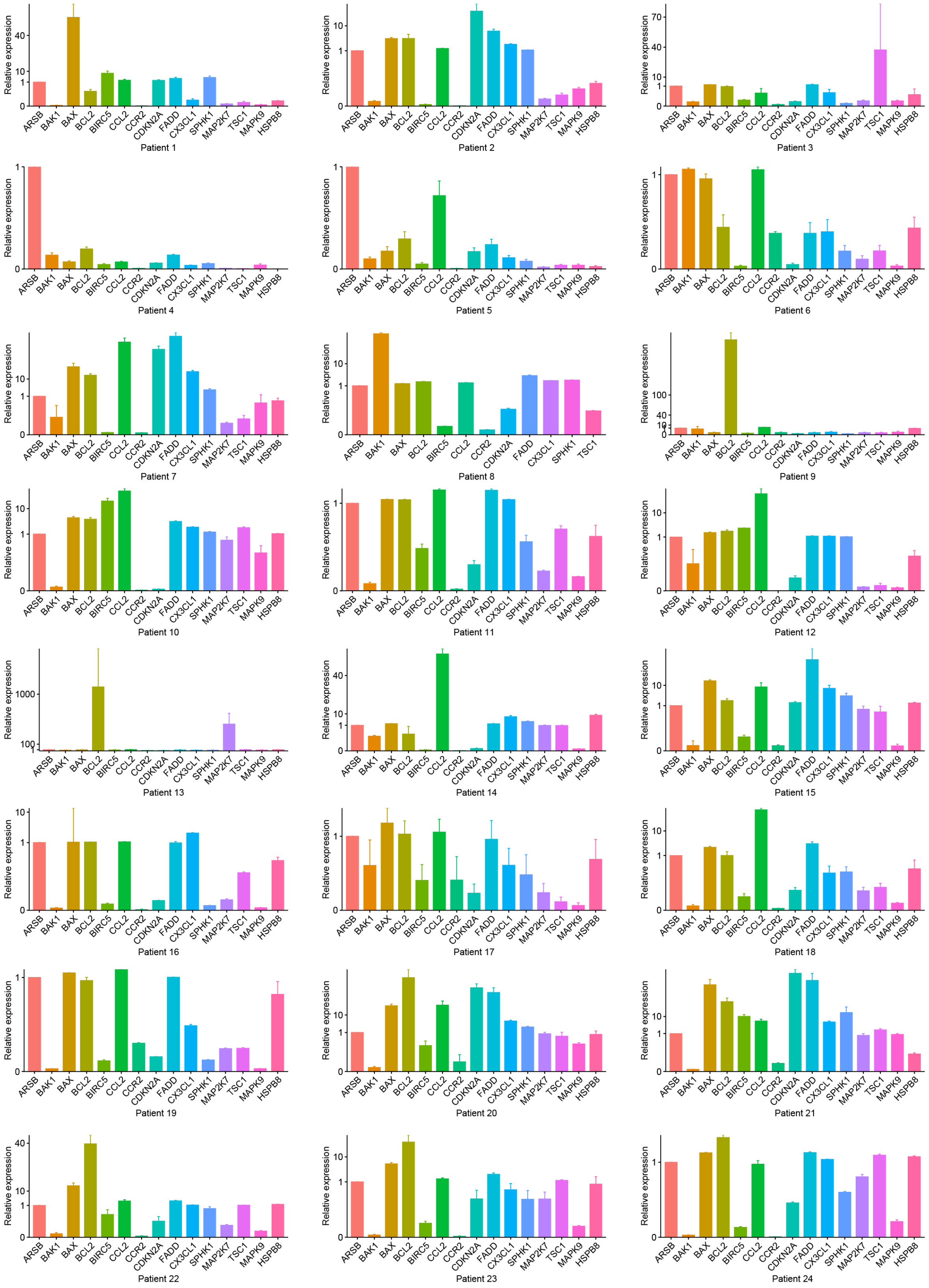
Relative expression
ARSB
BAK1
BAX
BCL2
BIRC5
CCL2
CCR2
CDKN2A
FADD
CX3CL1
SPHK1
MAP2K7
TSC1
MAPK9
HSPB8
Patient 1
Patient 2
Patient 3
Patient 4
Patient 5
Patient 6
Patient 7
Patient 8
Patient 9
Patient 10
Patient 11
Patient 12
Patient 13
Patient 14
Patient 15
Patient 16
Patient 17
Patient 18
Patient 19
Patient 20
Patient 21
Patient 22
Patient 23
Patient 24

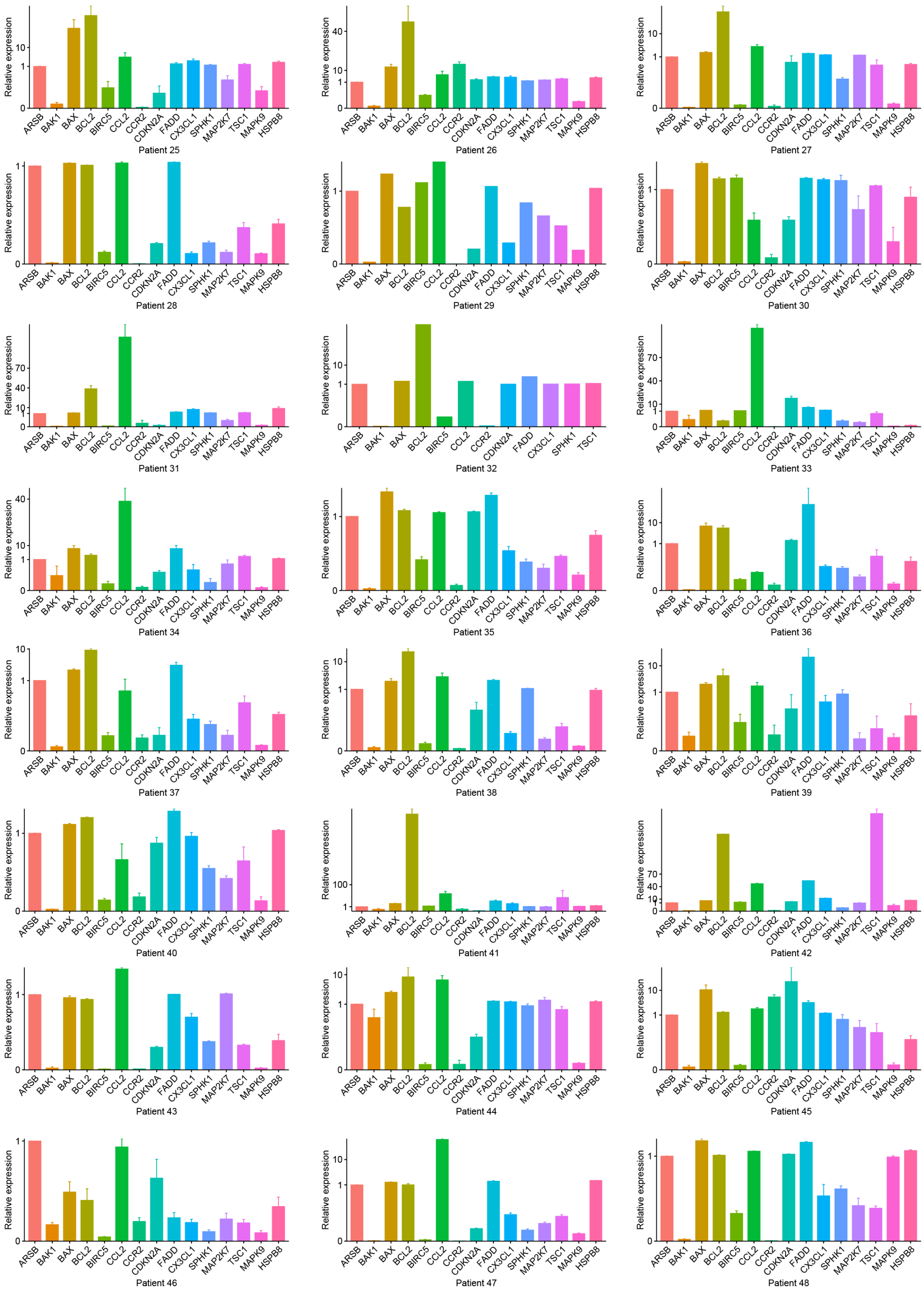
Relative expression
ARSB
BAK1
BAX
BCL2
BIRC5
CCL2
CCR2
CDKN2A
FADD
CX3CL1
SPHK1
MAP2K7
TSC1
MAPK9
HSPB8
Patient 25
Patient 26
Patient 27
Patient 28
Patient 29
Patient 30
Patient 31
Patient 32
Patient 33
Patient 34
Patient 35
Patient 36
Patient 37
Patient 38
Patient 39
Patient 40
Patient 41
Patient 42
Patient 43
Patient 44
Patient 45
Patient 46
Patient 47
Patient 48

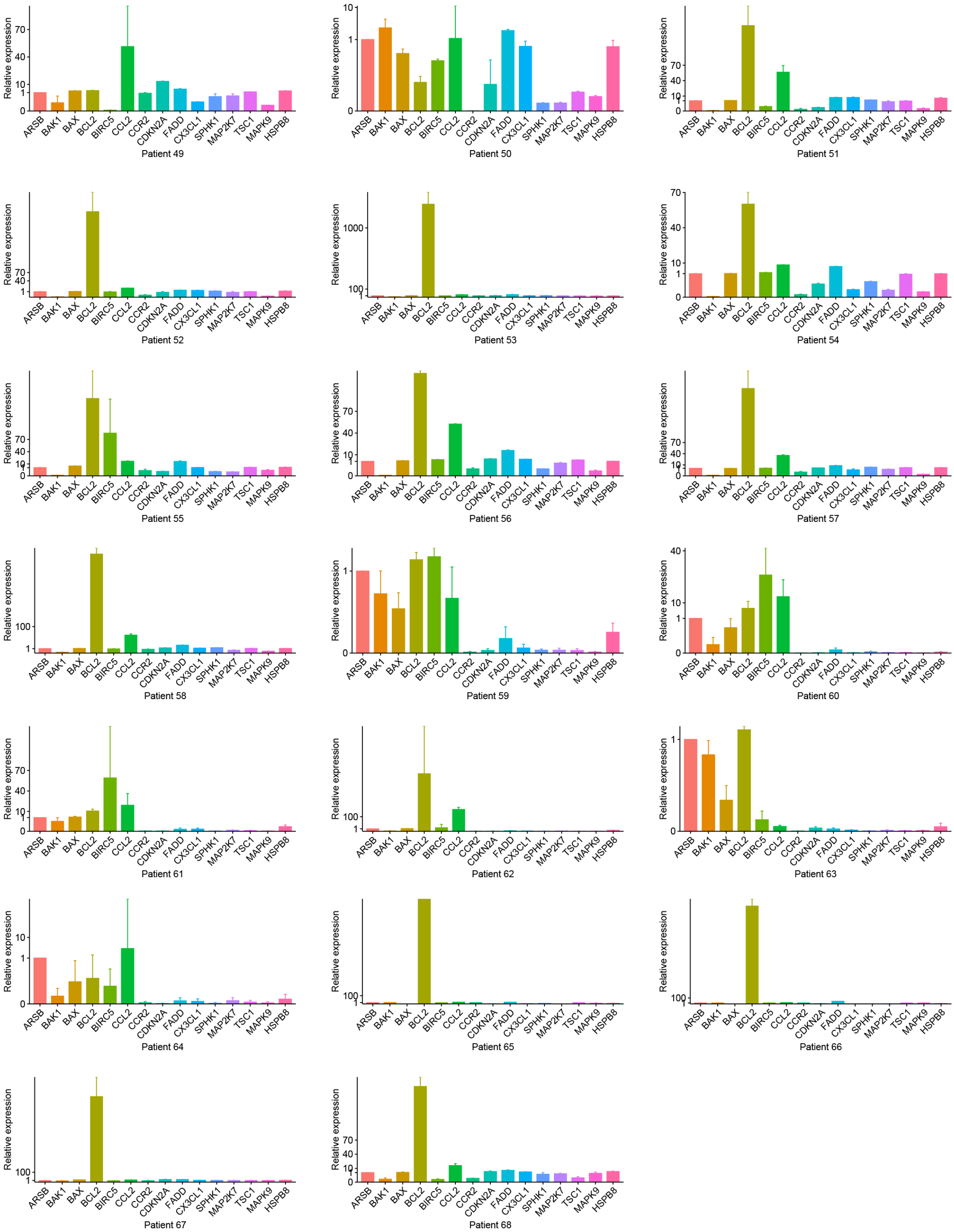
Relative expression
ARSB
BAK1
BAX
BCL2
BIRC5
CCL2
CCR2
CDKN2A
FADD
CX3CL1
SPHK1
MAP2K7
TSC1
MAPK9
HSPB8
0
1
10
40
70
100
1000
Patient 49
Patient 50
Patient 51
Patient 52
Patient 53
Patient 54
Patient 55
Patient 56
Patient 57
Patient 58
Patient 59
Patient 60
Patient 61
Patient 62
Patient 63
Patient 64
Patient 65
Patient 66
Patient 67
Patient 68
